# Affordable primary health care in low-income countries: can it be achieved?

**DOI:** 10.4102/phcfm.v2i1.246

**Published:** 2010-12-06

**Authors:** Dorothy E. Logie, Mike Rowson, Noleb M. Mugisha, Barbara Mcpake

**Affiliations:** 1 Scotland Rwanda Development Coalition, United Kingdom; 2 Centre for International Health and Development, UCL Institute of Child Health, United Kingdom; 3 Department of Family Medicine, Makerere University College of Health Sciences, Uganda; 4 Institute for International Health and Development, Queen Margaret University, United Kingdom

## FINANCING PRIMARY HEALTH CARE

Health systems in low income countries should be about equity and solidarity of health care provision for both urban and rural populations and about investing resources wisely. Instead, they are seriously under-funded, buffeted by multiple, disease-orientated programmes, or left to drift towards unregulated commercialism.^1^ Currently, 70% of health costs in resource poor countries are spent on 30% of the population,^2^ mainly on hospital and specialist care. It has been shown that countries with well-functioning primary health care (e.g. Thailand, Brazil, Cuba and Oman) have better health outcomes at low costs^1^ and, if they work in conjunction with first-referral hospitals, it is thought they can manage about 90% of their health demands.

The World Health Report 2008 re-asserts the guiding principles of the Alma Ata Declaration:

1. equity
2. inter-sectoral collaboration
3. access to essential drugs
4. cost-effectiveness
5. appropriate health technology
6. comprehensive care.^1^

It promotes primary health care, responsive to both individual and community needs and calls for universal coverage in the face of rising chronic and acute diseases. However, the reality is that inequities are deep and intractable. A crucial aspect in improving equity is ensuring cross-subsidy from higher to lower income groups and from low-risk to high-risk groups in the financing of the health system.^2^

Financing mechanisms for health will differ in each country and there is no ‘one-size-fits-all’ approach. However, the public sector has a key role to play in all health systems in planning the financing and provision of health care and in ensuring the regulation of what are often highly marketised health systems – all with the objectives of cost and quality control and the promotion of equity.^2^ In resource-poor countries today, financial systems are fragmented by the involvement of many different agencies (donors, insurers, government, faith-based organisations, non-governmental organisations [NGOs]) each with their own funding mechanisms. This reduces the possibility of universal risk-sharing and cross-subsidy. 

### Can the blocks and biases against financing primary health care be removed?

#### 1. No, if out-of-pocket payments continue to prevail

At present, for 5.6 billion people in low- to middle-income countries, half of all health expenditure is through out-of-pocket payments.^1^ These fees (or co-payments) made at the time of illness depend on the ability of the patient to pay and punish the poor, who often pay a larger proportion of their income for health than the rich. There is evidence that higher levels of out-of-pocket payment are associated with exclusion from health facilities altogether, with the ignoring of early disease and with higher levels of ‘catastrophic’ health expenditure, implying that long-term household prosperity may be affected.^2^ User fees are charged by both hospital and primary care providers and direct payments are also made to traditional healers and informal drug sellers. One study in Tanzania showed that a fatal illness in one member of the family could cost 64% of household income over a 6-months period.^3^ Even if exemptions do exist for care in the public sector, most countries do not have the administrative capacity to implement a reliable scheme.^4^ Although some countries (e.g. Ghana, South Africa, Uganda and Zambia) have abolished user charges at primary care level in the public sector (sometimes using money freed from debt repayments) informal or ‘under-the-counter’ payments may remain. Current opinion is that user fees must be kept to an absolute minimum, or be abolished, especially in rural areas.

#### 2. No, if donor funds cannot be targeted more effectively

Donor funding, potentially, can support the health policy priorities of national governments through basket funding at the budget or sector level − also known as a Sector Wide Approach (SWAp). A SWAp has the advantage of shifting the dialogue between government and donors up a level: from the planning and management of projects, to overall policy and financial frameworks. However, the jury is out on whether SWAps shift resources towards primary care or increase government ownership substantially. It also appears that, despite a policy emphasis on basket funding, the level of funding channelled through projects remains very high. This makes it important to support the ‘15by2015’ campaign launched by WONCA in March 2009, which calls for 15% of the funding of vertical programmes to be redirected to primary health care by 2015.^5^

#### 3. No, if the internal and external brain drain from government service continues

Budgets and health staff tend to revert to hospitals and central institutions as a result of the powerful interests of medical staff, the economic power of urban elites and the importance of the private sector. If internationally funded programmes operating separately from the government health service (e.g. HIV/AIDS treatment programmes) continue to pull staff from more generalised health system roles by offering high salaries and better working conditions, this will also continue to impoverish primary and rural health care and divert funds not only from staff costs, but also from training, supporting and monitoring of primary care workers.

#### 4. No, if national governments do not improve their commitment to primary care

The blocks and biases against financing primary health care cannot be removed if national governments do not improve their commitment to primary care, in order to counteract the considerable bias towards secondary/tertiary, high-tech health care, where 70% of health costs are spent on 30% of the population, predominantly the urban wealthier. Increased government spending on health directed to primary care is an essential component of this commitment.

Health care financing is one of Africa’s greatest challenges, as the tax base is low and unemployment is high. Publicly financed care allows for direct planning and targeting of resources and for the cross-subsidies required for equity. No African country has yet reached the target of 15% of total government budget for health, as pledged by the African heads of state at Abuja on 25 April, 2000.^6^ Annual budgets of US$20 per capita, or fewer, constrain planning. It has been suggested that in many countries, the tax base could be raised to at least 20% of GDP and from that base to increase public health expenditure, targeted to primary care (see Box 1).

All investments should emphasise improvement in the local capacity to manage resources for primary care. This also implies taking a robust stance against corruption and the diversion of resources to serve non-public interests, improving transparency, inspiring public trust and executing budget plans that are sometimes woefully under spent in relation to primary health care.^7^

### Are there ways of promoting and financing primary health care which work?

#### 1. Yes, if financing mechanisms that pool risk are employed

Financing mechanisms that pool risk across large populations include both social health insurance and public purse-based funding. Social health insurance demands good administrative skills. Heavily Indebted Poor Countries (HIPC) debt initiative funds have enabled increased and better-targeted primary health care provision in Uganda and Zambia.^8^ Ghana and Tanzania have been taking steps towards social health insurance schemes in the last decade.^9^ There are drawbacks when a large part of the population works in the informal sector. Selective insurance (cream-skimming) may exclude the seriously ill. Payments are usually income-related and therefore progressively (the rich contribute more than the poor) but if a flat rate is charged, the opposite is true. However, social (compulsory) schemes have several cost and equity benefits over private health insurance. Ideally, social insurance schemes should cover preventative and curative service but more often they only deal with curative service and acting through a fee-for-service payment system, they may increase over-prescribing.

#### 2. Yes, if community-based health insurance schemes are used to mobilise financial resources for health with pre-payment and risk sharing as components

Rwanda has shown that the use of the health service has increased dramatically since the introduction in 1999 of the Mutuelle de Sante.^9^ This independent, country-wide community insurance scheme now covers 80% of the population and most of the primary and secondary care. The scheme is run as an autonomous organisation (parastatal), managed by its members pooling risk at the village and district levels. Each member of the scheme contributes 1000 RWf (US$2) annually and also pays a 10% fee for each illness episode. Decisions relating to the scheme are made through an elected village committee that decides who is too poor to pay. These exemptions are then paid for by donors. Estimates suggest that between 15% and 30% of the population may need to have fees waived. The village committees also monitor the quality of care.^9^ This is an innovative approach.

The funding base of these types of schemes may be fragile and fragmented if the funding pools are small and may be prone to collapse. To avoid this, it is important that they cover a large population base so that the scope for cross-subsidy is large and it is important that the state and donors are involved in supporting community processes and subsidising the exemptions. Income-rating of contributions is unusual.

#### 3. Yes, if we concentrate on the relationships between patients, professionals and money

Health care is centred on human interaction. Yet it is surprising how little this is mentioned when we talk about the prospects for improving primary health care. Whilst money is vital, it is equally important to focus on how the care is delivered. Trust in public services may be reignited and out-of-pocket payment avoided if improved quality of care were on offer, with supportive teams led by trained family medicine physicians. Complaints abound about the quality of care in low-income contexts, with absenteeism, poor and abusive care, as well as bribes and mis-charging being commonplace. The poor suffer most from this abuse: at the bottom of the social hierarchy they are more likely to face bullying and discriminatory behaviour.^10,11^

A key step is for governments to focus on the relationship between patients and health care providers. Monitoring of care, through patients and consumer groups (perhaps funded by the state) and the involvement of lay people at different levels of health systems, may improve both quality of care and the accountability of the system as a whole. Improvement will happen when patients know what they are entitled to by establishing well-publicised minimum standards which are raised over time.^12^ One of the problems with heavily donor-subsidised forms of health care is that the size of the subsidy makes governments focus more on their accountability and relationships with donors instead of their relationship between their own citizens and the state, to help improve the behaviour of health providers in both public and private sectors and to combat corruption and malfeasance through better public oversight.^13^

## CONCLUSIONS

### Can affordable primary health care be achieved?

Box 1 suggests some new Millennium Development Goals, targeted at financing health care. For investment in primary care to be successful, it must stimulate a lasting change to meet universal health needs. Decentralised, flexible decision-making must be encouraged. Even if finances are in place, there remain considerable non-financial barriers to accessing primary health care for the poor (e.g. geographical isolation, culture, opportunity costs of seeking care and gender barriers). Sustainability and universal coverage is the litmus test.

The ‘15 by 2015’ campaign^5^ could bring significant extra funding for primary care if accepted by the big donors. The WHO, too, must give strong leadership, with a worldwide plan for primary care by creating a specific, high-level unit to examine costs, quality and staffing required. It has already shown the initiative1 in promoting the type of care that puts people first, that is, primary health care. This is needed now, more than ever.

**BOX 1:**
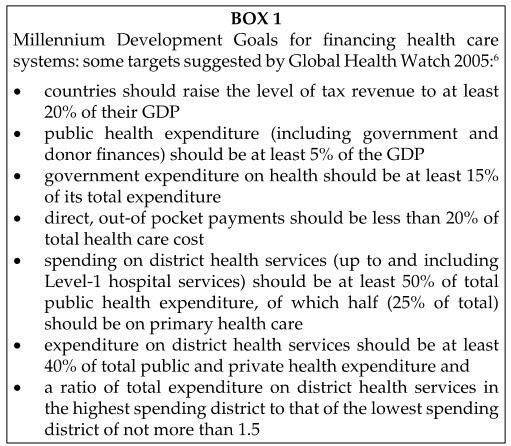
Millennium Development Goals for financing health care systems: some targets suggested by Global Health Watch 2005:^6^
